# A *Schistosoma japonicum *chimeric protein with a novel adjuvant induced a polarized Th1 immune response and protection against liver egg burdens

**DOI:** 10.1186/1471-2334-9-54

**Published:** 2009-05-06

**Authors:** Xindong Xu, Dongmei Zhang, Wei Sun, Qingfeng Zhang, Jingjing Zhang, Xiangyang Xue, Luhui Shen, Weiqing Pan

**Affiliations:** 1 Institute for Infectious Diseases & Vaccine Development, Tongji University School of Medicine, 1239 Siping Road, Shanghai 200092, PR China; 2 Department of Pathogen Biology, Second Military Medical University, 800 Xiang Yin Road, Shanghai 200433, PR China

## Abstract

**Background:**

Schitosomiasis japonica is still a significant public health problem in China. A protective vaccine for human or animal use represents an important strategy for long-term control of this disease. Due to the complex life cycle of schistosomes, different vaccine design approaches may be necessary, including polyvalent subunit vaccines. In this study, we constructed four chimeric proteins (designated SjGP-1~4) via fusion of Sj26GST and four individual paramyosin fragments. We tested these four proteins as vaccine candidates, and investigated the effect of deviating immune response on protection roles in mice.

**Methods:**

The immunogencity and protection efficacy of chimeric proteins were evaluated in mice. Next, the chimeric protein SjGP-3 was selected and formulated in various adjuvants, including CFA, ISA 206, IMS 1312 and ISA 70M. The titers of antigen-specific IgG, IgE and IgG subclass were measured. The effect of adjuvant on cytokine production and percentages of CD3^+^CD8^-^IFN-γ^+ ^cells and CD3^+^CD8^-^IL-4^+ ^cells were analyzed at different time points. Worm burdens and liver egg counts in different adjuvant groups were counted to evaluate the protection efficacy against cercarial challenge.

**Results:**

Immunization of mice with chimeric proteins provided various levels of protection. Among the four proteins, SjGP-3 induced the highest level of protection, and showed enhanced protective efficacy compared with its individual component Sj26GST. Because of this, SjGP-3 was further formulated in various adjuvants to investigate the effect of adjuvant on immune deviation. The results revealed that SjGP-3 formulated in veterinary adjuvant ISA 70M induced a lasting polarized Th1 immune response, whereas the other adjuvants, including CFA, ISA 206 and IMS 1312, generated a moderate mixed Th1/Th2 response after immunization but all except for IMS 1312 shifted to Th2 response after onset of eggs. More importantly, the SjGP-3/70M formulation induced a significant reduction in liver egg deposition at 47.0–50.3% and the number of liver eggs per female at 34.5–37.2% but less effect on worm burdens at only 17.3–23.1%, whereas no effect of the formulations with other adjuvants on the number of liver eggs per female was observed.

**Conclusion:**

Construction of polyvalent subunit vaccine was capable to enhance immunogenicity and protection efficacy against schistosomiasis. There was correlation of the polarized Th1 response with reduction of liver egg burdens, supporting the immune deviation strategy for schistosomiasis japonica vaccine development.

## Background

Schistosomiasis remains one of the most prevalent parasitic diseases in the world, affecting more than 200 million people in developing countries [1]. Of the three major schistosome species that infect humans, *Schistosoma japonicum *is recognized as the most difficult to control because of its zoonotic nature. Several types of important livestock, such as water buffaloes and domestic pigs, are main reservoir hosts of *S. japonicum*, and eggs in their feces are of prime importance for continued transmission of this parasite in humans. In the past five decades, schistosomiasis has been largely controlled in China through widespread treatment with the anti-schistosome agent, praziquantel, plus large-scale environmental campaigns to eradicate the intermediate host snail. However, there has been a resurgence of schistosomiasis in recent years in some provinces of China, due to the inability of chemotherapy to prevent new infection and difficulties associated with snail intermediate hosts eradication [2]. As a result, a protective vaccine for human or domestic animal use represents an important strategy for long-term control of schistosomiasis japonica [3,4].

Numerous antigens have been identified as schistosomiasis japonica vaccine candidates [5]. Among them, *S. japonicum *26 kDa glutathione S-transferase (Sj26GST) and paramyosin are two leading vaccine candidates. In the schistosome, GSTs are expressed in the parenchymal cells of male parasites and in the parenchymal cells between the vitelline glands in female worms [6,7]. They function to detoxify and remove harmful molecules from the organisms [8]. Vaccination of mice with Sj26GST provided a moderate level of protection [9]. Paramyosin is a 97 kDa myofibrillar protein with a coiled-coil structure that is widely distributed in *S. japonicum *at different life stages, including cercariae, lung-stage schistosomula, and in adult worms [6]. Immunization of animals with native and recombinant paramyosin conferred significant protection against challenge with *S. japonicum *[10,11]. In our previous study, we divided paramyosin into four overlapping fragments (Pmy-F1, Pmy-F2, Pmy-F3, and Pmy-F4) to evaluate the protective efficacy of each fragment. We found all four fragments of paramyosin produced similar levels of protection in mice [12].

Although both Sj26GST and paramyosin provide a certain level of protection against schistosome infection, neither has consistently provided sufficient levels of protection [4]. Due to the complex life cycle of schistosome, unconventional vaccine design approaches may be needed. Recently, a novel approach utilizing a polyvalent subunit vaccine has been successfully applied by our group to the PfCP-2.9 chimeric vaccine candidate against malaria, another parasite with a complex life cycle [13-15]. Such chimeric proteins are capable of enhancing immunogenicity and inhibiting parasite growth. Based on our successful experience with the polyvalent subunit malaria vaccine, we have now constructed four *S. japonicum *chimeric proteins, designated SjGP-1, SjGP-2, SjGP-3 and SjGP-4, comprised of the full-length Sj26GST sequence and Pmy-F1, Pmy-F2, Pmy-F3 or Pmy-F4, respectively. These chimeric proteins were tested in mice for immunogenicity and protective efficacy against challenge with *S. japonicum *in the current study.

Many studies highlight the importance of immunoregulatory role of cytokines associated with Th1/Th2 immune responses in both anti-parasite and anti-pathology immunity. Therefore, immune deviation has been considered a modern immunology strategy for schistosomiasis vaccine development [16]. Many factors were shown to influence immune response, but use of adjuvant would be a useful protocol for immune deviation [17]. In an attempt to increase the efficacy of chimeric protein constructed in this study and to investigate the effect of deviating immune response on protection roles, SjGP-3, the chimeric protein that gave the best protective efficacy in challenge experiment, was formulated with four different adjuvants (CFA, Montanide^® ^ISA 206, Montanide^® ^IMS 1312 and Montanide^® ^ISA 70M) and tested in mice. SjGP-3 emulsified with the veterinary adjuvant ISA 70M induced a strong polarized Th1 immune response after immunization and this SjGP-3-driven Th1 response could be maintained after cercarial challenge. However, the same antigen formulated with CFA or ISA 206 induced a moderate mixed Th1/Th2 immune response after vaccination, but this mixed immune response shifted to Th2 immune response after parasite maturation and onset of egg production. Importantly, the SjGP-3-inducing polarized Th1 immune response was significantly associated with the reduction of liver egg burdens as well as the number of liver eggs per female worm.

## Methods

### Parasites and animals

A mainland strain of *Schistosoma japonicum *used for all experiments was originally maintained in *Oncomelania hupensis *snails and in New Zealand White rabbits. Six to 8-week-old BALB/c mice were purchased from the Songjiang Animal Facility of the Chinese Academy of Sciences of Shanghai. Seven weeks after cercarial challenge, adult worms were recovered by perfusion of infected rabbits and homogenized in cold buffer containing 140 mM NaCl, 2.7 mM KCl, 10 mM Na_2_HPO_4_, 1.8 mM KH_2_PO_4_, 2 mM phenyl methyl sulfonyl fluoride (PMSF). Soluble worm antigen products (SWAP) were obtained from adult worms following sonication and centrifugation [18]. To purify the native Sj26GST of adult worm, SWAP was applied to the Glutathione Sepharose 4B column (Amersham Bioscience, USA). The column was then washed with PBS and the bound material was eluted with elution buffer containing 10 mM Glutathione in 50 mM Tris-HCl, pH8.0.

### Construction of chimeric proteins

The amino acid sequence of paramyosin of *S. japonicum *mainland strain (Genbank accession number: AAD29285) was translated into a DNA sequence using yeast (*Pichia pastoris*) codon usage [19]. The resulting DNA sequence was divided into four fragments that were synthesized separately as described in our previous study [20]. The individual synthesized fragment was inserted into the vector pGEX-4T-1 via *Bam*HI and *Eco*RI restriction sites in frame with the coding sequence of Sj26GST which was located on the vector so that individual chimeric protein consisting of Sj26GST and individual paramyosin fragment. The sequence of each chimeric protein was then generated by PCR using the upstream primer, P0: 5'-GC CTC GAG AAA AGA ATG TCC CCT ATA CTA GGT-3', and four different downstream primers, P1: 5'-CC GAA TTC CTA TTA GTG ATG ATG GTG GTG ATG GAA ACG ATT TCT AGA TTC ATC-3'; P2: 5'-CC GAA TTC CTA TTA GTG ATG ATG GTG GTG ATG GGA ACG CAA AGC TTC CAG GTC-3'; P3: 5'-CC GAA TTC CTA TTA GTG ATG ATG GTG GTG ATG GGC TTG CAT GAC GCC AAT GTC-3'; P4: 5'-CC GAA TTC CTA TTA GTG ATG ATG GTG GTG ATG CAT CAT AGA TGT AGC TCT CAT AC-3'. The resulting PCR products were first inserted into the vector pPIC9, respectively, and then each *Sac*I/*Sal*I fragment was excised from the recombinant pPIC9 plasmids and inserted into the *Pichia *expression vector, pPIC9K. To facilitate purification, 6 × His tags were included at the C-terminus of the chimeric proteins.

### Expression of the chimeric genes in either *Pichia pastoris *or *E. coli*

The recombinant pPIC9K plasmids were linearized before transfered by electroporation into *Pichia pastoris *GS115. Selection of His^+ ^transformants and G418-resistant clones was carried out according to the manufacturer's instruction manual (Invitrogen, USA). The selected clones were first cultured in 3 ml Minimal Glycerol Medium at 30°C overnight until the OD (600 nm) reached to 2–6. Cells were then grown in 3 ml Buffered Methanol-complex Medium containing 0.5% (v/v) methanol for induction of protein expression. Methanol was added to the culture at 24 h intervals to maintain the final concentration at 0.5% (v/v). To express the protein in a 15-L fermentor, a 250 mL culture of yeast cells was first grown at 30°C for 22 h in Minimal Glycerol Medium and then inoculated into the fermentor containing 6000 mL of minimal salts fermentation medium. The cells were grown at 30°C and harvested at 48 h after methanol induction.

For those fusion proteins that failed to be expressed in *Pichia*, the recombinant plasmids based on pGEX-4T-1 were transformed into *E. coli *BL21 (DE3) strain. The selected clones were grown first in LB/Amp^+ ^at 37°C for 3 h. Then IPTG was added at 0.5 mM to induce protein expression at 25°C for 5 h in shake flasks.

### Purification of recombinant proteins

To purify the recombinant proteins produced in *P. pastoris*, the fermentation supernatant was dialyzed extensively against dialyzing buffer containing 20 mM sodium phosphate, 0.5 M NaCl, 20 mM imidazole, pH 7.4. To purify the recombinant proteins produced in *E. coli*, cells were harvested and disrupted by sonication in the dialyzing buffer described above. The dialyzed fermentation supernatant or *E. coli *cell lysate supernatant material was applied to a Ni-NTA column (Qiagen, Germany) for purification. Further purification was carried out on a Glutathione Sepharose 4B column (Amersham Bioscience). LPS contamination in purified proteins was excluded by *Limulus *lysate test (SIGMA, USA). Protein concentration was determined by the Bradford method [21].

### Immunization schedule

Mice were injected s. c. with 20 μg of recombinant fusion proteins or Sj26GST in 50 μl PBS formulated with an equal volume of complete Freund's adjuvant (CFA, SIGMA) or incomplete Freund's adjuvant (IFA, SIGMA) at 3-week intervals. CFA was used for the initial immunization and IFA for the two subsequent boosts. The control group was subjected to the same immunization schedule, but PBS replaced the fusion proteins.

In the experiment to evaluate the protective efficacy of one fusion protein mixed with different adjuvants, the recombinant fusion protein that produced the best protective efficacy, SjGP-3, was selected as the antigen. Four different adjuvants were tested separately: CFA, ISA206, IMS1312 and ISA 70M. Mice were injected s. c. with 20 μg SjGP-3 protein in PBS formulated with CFA, ISA206, IMS1312 or ISA 70M, respectively. The formulations of ISA 206, IMS 1312 and ISA 70M were issued by SEPPIC (France). Briefly, the antigen was added in ISA 206, IMS 1312 and ISA 70M at a ratio of 46:54, 50:50 and 30:70 (m/m), respectively. The formulations were stirred for 15 to 30 min at 2000 rpm. Except for CFA, the adjuvants were also used for the two subsequent boosts at 3-week intervals. The control group was injected with PBS instead of fusion protein and adjuvants. The experiment was repeated twice.

### Immunoblotting

The purified native Sj26GST or SWAP was separated by SDS-PAGE and then transferred onto a 0.45 μm of PVDF membrane using a Bio-Rad protein transfer unit. Membranes were probed with sera from immunized mice, incubated with goat-anti-mouse IgG-AP, and revealed by NBT-BCIP detection system (SIGMA).

### Measurement of antigen-specific antibody

Serum samples were collected before injection and on week 2, 5 and 8 after the initial immunization. Enzyme-linked immunosorbent assay (ELISA) was performed to detect the levels of different isotypes against the fusion or individual proteins using HRP-labeled goat antibodies specific for IgG, IgE and IgG subclasses (Bio-Rad, USA). ELISA was performed as described before [12]. Cut-off values were determined from the mean plus three standard deviations from the pre-immunization sera.

### Quantification of IL-4 and IFN-γ

On week 9 (before challenge), 12(three weeks after cercarial challenge) and 15 (six weeks after cercarial challenge), spleen cells from individual mice were cultured at 5 × 10^6 ^cells/well in 24-well plates (Corning, USA) in RPMI 1640 medium (Gibico, USA) supplemented with 10% fetal calf serum (SIGMA). Anti-CD3 (1 μg/ml), recombinant protein (30 μg/ml) or medium was used to stimulate splenocytes. At 72 h after stimulation, supernatants were collected for cytokine analysis. The production of IL-4 and IFN-γ were measured by ELISA as described [22,23] by cytokine-specific Abs, recombinant cytokine standards, biotinylated detection Abs, and avidin-HRP (eBioscience, USA).

### Intracellular cytokine detection by flowcytometry

Splenocytes (2 × 10^6 ^cells/ml) were harvested from mice immunized with SjGP-3 formulated with various adjuvants on week 9, 12 and 15, and stimulated with 25 ng/ml phorbol myristate acetate (PMA), 1 ug/ml ionomycin plus 1 ug/ml brefeldin A (BFA) (SIGMA) at 37°C for 6 h on a 24-well plate (Corning). Splenocytes were stained extracellularly with PE-Cy5 conjugated anti-CD3 and FITC conjugated anti-CD8 (eBioscience). After fixation in FIX&Perm Medium A (CALTAG, USA), cells were stained intracellularly with PE conjugated Rat IgG1 (isotype control), anti-mouse IFN-γ antibody or anti-mouse IL-4 antibody (eBioscience) in FIX&Perm Medium B (CALTAG). A FACScan flow cytometer with Cell Quest software was used for data acquisition and analysis.

### Cercarial challenge and determination of resistance

On week 9, mice were challenged with cercariae. Mice were fixed in wooden plates and the hairs on the abdomen were shaved by an animal clipper. The shaved part was then wetted with dechlorine tap water. Each mouse was infected with 40 ± 2 cercariae, which were placed on a coverglass (12 × 12 mm) with the aid of a capillary dropper under microscope. The coverglass was then placed up side down on the shaved area for at least 20 minutes.

On week 15, adult worms were recovered by perfusion from the mesenteric vein. Livers were weighed and processed as described previously [12]. Briefly, the livers were digested with 4% KOH overnight, and the number of eggs was determined by microscopic examination. Total adult worm burdens and liver eggs were counted. The level of protection of vaccinated group was calculated as a percentage based on the reduction in worm burdens or liver eggs per gram of liver, compared with those from the control group. The reduction rate of liver eggs per female worm was also calculated according to the following formula: (1-mean number of liver eggs per female worm in immunized mice/mean number of liver eggs per female worm in control mice) ×100.

### Statistical analysis

Data of worm burdens and egg counts were expressed as the mean ± standard error. The other data were expressed as the mean ± standard deviation. Multiple comparisons were analyzed by a LSD one-way ANOVA test, with a *p*-value < 0.05 considered significant. We used SPSS version 13 for all statistical analyses.

## Results

### Construction and expression of chimeric genes

**Figure 1:**
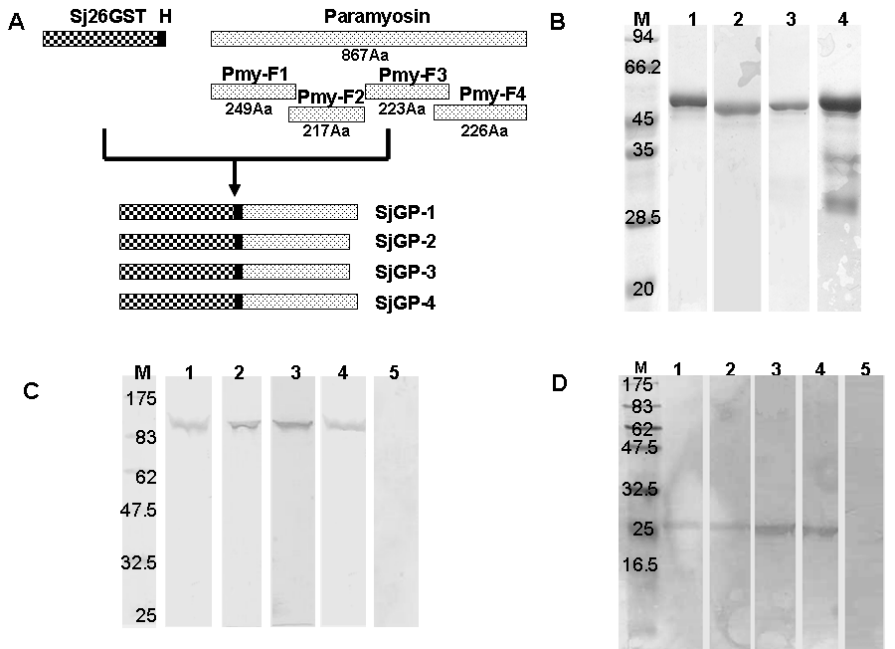
**Schematic representation of chimeric gene constructs, recombinant proteins and interaction of immune sera with native proteins**. **(A) **Schematic representation of SjGP-1, SjGP-2, SjGP-3, and SjGP-4 gene construction. Paramyosin was divided into four overlapping fragments and the length of amino acid (Aa) sequence of each fragment is indicated. Each paramyosin fragment was fused to Sj26GST to generate chimeric gene via a hinge (H) consisting of six amino acid residues that were the thrombin recognition site. **(B) **Purity analysis of purified chimeric proteins by SDS-PAGE. Lane M: molecular mass maker; lanes 1 to 4: purified SjGP-1, SjGP-2, SjGP-3, and SjGP-4 protein, respectively. **(C) **Interaction of immune sera against the individual chimeric proteins with soluble worm antigen preparation (SWAP) by Western Blot. The SWAP material was separated by SDS-PAGE and analyzed by Western Blot using either immune sera to the individual chimeric proteins or negative control sera. Lane M: molecular mass maker; lanes 1 to 4: sera of mice immunized with SjGP-1, SjGP-2, SjGP-3, and SjGP-4, respectively; lane 5: serum from PBS control mice. **(D) **Interaction of immune sera against the individual chimeric proteins with purified native Sj26GST. The purified native Sj26GST was separated by SDS-PAGE and analyzed by Western Blot using either immune sera to individual chimeric protein or negative control sera. Lane M: molecular mass maker; lanes 1 to 4: sera of mice immunized with SjGP-1, SjGP-2, SjGP-3, and SjGP-4, respectively; lane 5: serum from PBS control mice.

Four overlapping fragments derived from *S. japonicum *paramyosin were generated in our previous study using codon optimization and gene synthesis. The chimeric proteins were constructed by fusing paramyosin fragments with Sj26GST gene, via a hinge of six amino acid residues that was the thrombin recognition site (Figure 1A). The resulting fusion fragments were then inserted into the vector pPIC9K for expression in *Pichia*. After methanol induction, only the SjGP-2 protein was detected in supernatant of the yeast culture; no other constructs were detected in this system. Alternatively, the remaining three genes (SjGP-1, SjGP-3 and SjGP-4) were successfully produced in soluble form after induction by 0.5 mM IPTG at 25°C in *E. coli*.

To facilitate purification of protein, a 6 × His tag was incorporated at the C-terminus of each construct, allowing isolation of recombinant protein on a Ni-NTA column. Because each construct contained Sj26GST, it was also possible to isolate the chimeric proteins by Glutathione Sepharose 4B column. Thus, all of the recombinant proteins were purified by combining the purification processes of Ni-NTA with Glutathione Sepharose 4B affinity chromatography. The purities of proteins were close to 95% as determined by SDS-PAGE, with the exception of SjGP-4, in which a degradation product was observed (Figure 1B) and confirmed by Western blotting using mouse immune sera against Sj26GST or Pym-F4, retained in our lab (data not shown).

### Immunogencity of the chimeric proteins

The amount of IgG in serum collected on week 8 was determined by ELISA. Antibody titers were measured against purified fusion proteins (SjGP-1 to SjGP-4) and individual components (Sj26GST and the corresponding paramyosin fragments Pmy-1 to Pmy-4). High levels of antigen-specific antibodies were elicited by all fusion proteins after three times immunization. Of these, SjGP-1 and SjGP-3 induced the highest levels of antibodies at titers of 1/1,310,000 and 1/1,231,000, respectively. Moreover, the antibodies to chimeric proteins recognized both of the individual components, Sj26GST and the corresponding paramyosin fragments (Table 1). To investigate whether the antibodies induced by the recombinant chimeric proteins can recognize native paramyosin and Sj26GST, soluble worm antigen products (SWAP) were probed by Western blotting using mouse anti-sera obtained after the third vaccination. As shown in Figure 1C, antibodies to chimeric proteins bound to bands at 97 kDa (Figure 1C, lanes 1–4), whereas no specific protein band was detected using pre-immune sera (Figure 1C, lane 5). However, the 26 kDa Sj26GST protein was not detected in this preparation, perhaps due to low level expression of this protein *in vivo*. Therefore, the native Sj26GST was first concentrated from SWAP by a purification process using Glutathione Sepharose 4B column. Then the purified product was tested. The protein bands at 26 kDa were consistently detected by immune sera (Figure 1D, lanes 1–4).

**Table 1:** Antibody titers, worm and egg burdens, protective efficacy in mice vaccinated with schistosome chimeric proteins

Groups^a^	IgG Titers^b^		Worm burden	%Worm reduction	Liver eggs (epg)	% Egg reduction
			Mean ± SE		Mean ± SE ^c^	
SjGP-1	To SjGP-1	1,310,000	25.8 ± 1.3	27.7*	25,900 ± 2,124	27.7*
	Pmy-F1	804,000				
	Sj26GST	112,000				
SjGP-2	To SjGP-2	550,000	34.0 ± 1.6	4.2	32,100 ± 2,150	10.3
	Pmy-F2	63,000				
	Sj26GST	19,000				
SjGP-3	To SjGP-3	1,231,000	22.2 ± 1.6	37.5*^,#^	26,700 ± 2,011	25.4*
	Pmy-F3	663,000				
	Sj26GST	142,000				
SjGP-4	To SjGP-4	804,000	28.3 ± 1.9	20.7*	30,000 ± 3,291	16.2
	Pmy-F4	136,000				
	Sj26GST	23,000				
Sj26GST	To Sj26GST	230,000	30.8 ± 1.4	13.7	28,100 ± 1,524	21.5*
Control	To all above	500	35.7 ± 2.5	/	35,800 ± 3,178	/

^a^: The number of mice per group is from 9 to 10; ^b^: "Pmy-F1" to "Pmy-F4" represents the four paramyosin fragments, respectively. Cut-off value was determined based on the pre-immunized geometric mean and three standard deviations. ^c^: Number of liver eggs was calculated as egg numbers per gram of liver (epg). *: *p *< 0.05 when compared with PBS control group. ^#^: *p *< 0.05 when compared with Sj26GST group. Each value was derived from the arithmetic means of individuals in the group.

### Protection efficacy against cercarial challenge

Mice were infected percutaneously with 40 cercariae of *S. japonicum *by the cover method (as described in Method). Mice were sacrificed on week 15 (six weeks after a challenge infection). Adult worms were recovered by perfusion from the mesenteric vein and liver eggs were counted. As shown in Table 1, compared with control group, worm burdens of vaccinated mice were reduced (P < 0.05) by 27.7%, 37.5% and 20.7% for SjGP-1, SjGP-3 or SjGP-4, respectively. SjGP-2 vaccination did not reduce worm burdens (4.2%, P > 0.05). More importantly, fusion protein SjGP-3 enhanced protection against worm burdens compared individual component of Sj26GST (P < 0.05). In addition, significant reductions of liver egg counts were observed in SjGP-1 and SjGP-3 groups, but not in SjGP-2 and SjGP-4 groups compared with control group (Table 1). Notably, similar level of egg reduction but much higher worm reduction was observed in SjGP-3 group compared with SjGP-1. Because of this, SjGP-3 was selected for further investigation as a vaccine candidate against shistosomiasis.

### Immunogenicity of SjGP-3 formulations with various adjuvants

**Figure 2:**
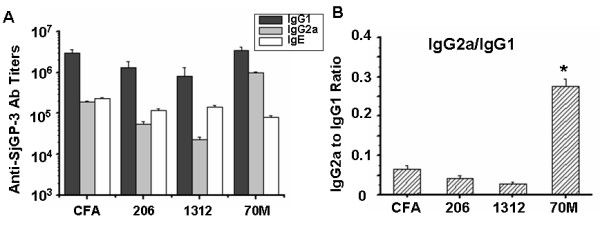
**SjGP-3-specific immunoglobulin subtypes (IgG1, IgG2a and IgE)**. Groups of mice were immunized with SjGP-3 formulations. Serum was collected after three time immunization. IgG1, IgG2a and IgE titers were detected by ELISA in triplicate wells respectively and the IgG2a to IgG1 ratio was calculated. **(A) **Anti-SjGP-3 antibody titers. **(B) **IgG2a/IgG1 ratio. * *p *< 0.05 when compared with other groups. This picture shows one experiment representative of two independent trials with similar results.

To investigate the effect of adjuvant on immunogenicity of the chimeric protein, SjGP-3 was formulated in various adjuvants, including complete Freund's adjuvant (CFA), ISA 206, IMS 1312 and ISA 70M (SEPPIC, France). Mice were immunized s.c. with 20 μg recombinant proteins. As shown in Table 2, similar levels of total IgG were generated in CFA, ISA 206 and ISA 70M adjuvant groups while ISM 1312 adjuvant produced much lower levels of antibody. Further analysis of the IgG isotypes and IgE revealed that IgG1 was the predominant subclass in all of the adjuvant groups. However, high levels of IgG2a were observed in the protein groups formulated with ISA 70M that was 43, 18 and 5-fold higher than ISM 1312, ISA 206 and CFA formulations respectively, while the titer of IgE in ISA 70M group was only1/10, 3/10 and 7/10 of that in CFA, ISA 206 and IMS 1312 group respectively (Figure 2A). The ratio of IgG2a/IgG1 in ISA70M group was the highest compared to other groups (Figure 2B). These results were repeatable in the second experiment. The data suggested that distinctive types of immunity were induced by the different adjuvants.

**Table 2:** Effects of immunization with the SjGP-3 formulated by various adjuvants on induction of IgG and protective efficacy against challenge in mice

Groups ^a^		IgG titers ^b^	Worm burdens Mean ± SE	%Worm reduction	Liver eggs (epg) Mean ± SE ^c^	% Egg reduction
Exp1	SjGP-3/CFA	1,890,000	19.8 ± 0.9	41.1*	20,900 ± 1,947	26.6*
	SjGP-3/ISA206	1,290,000	23.4 ± 1.4	30.4*	21,200 ± 2,307	25.6*
	SjGP-3/IMS1312	680,000	25.3 ± 1.2	24.7*	24,400 ± 2,604	14.4
	SjGP-3/ISA70M	1,790,000	27.8 ± 1.2	17.3*	15,100 ± 1,578	47.0*^,#^
	Control	< 500	33.6 ± 1.0	/	28,500 ± 2,777	/
Exp2	SjGP-3/CFA	1,831,000	20.9 ± 1.2	38.2*	26,900 ± 2,557	29.6*
	SjGP-3/ISA206	1,160,000	24.0 ± 1.6	29.0*	28,300 ± 1,808	25.9*
	SjGP-3/IMS1312	783,000	24.7 ± 1.9	26.9*	31,500 ± 2,593	17.5
	SjGP-3/ISA70M	1,841,000	26.0 ± 0.9	23.1*	19,000 ± 1,817	50.3*^,#^
	Control	< 500	33.8 ± 0.8	/	38,200 ± 3,428	/

^a^: The number of mice per group is from 8 to 10; ^b^:Cut-off value was determined based on the pre-immunized geometric mean and three standard deviations. ^c^: Number of liver eggs was calculated as egg numbers per gram of liver (epg). *: *p *< 0.05 when compared with PBS control group. ^#^: *p *< 0.05 when compared with other adjuvant groups. Each value was derived from the arithmetic means of individuals in the group.

### Effect of adjuvant on cytokine production

**Figure 3:**
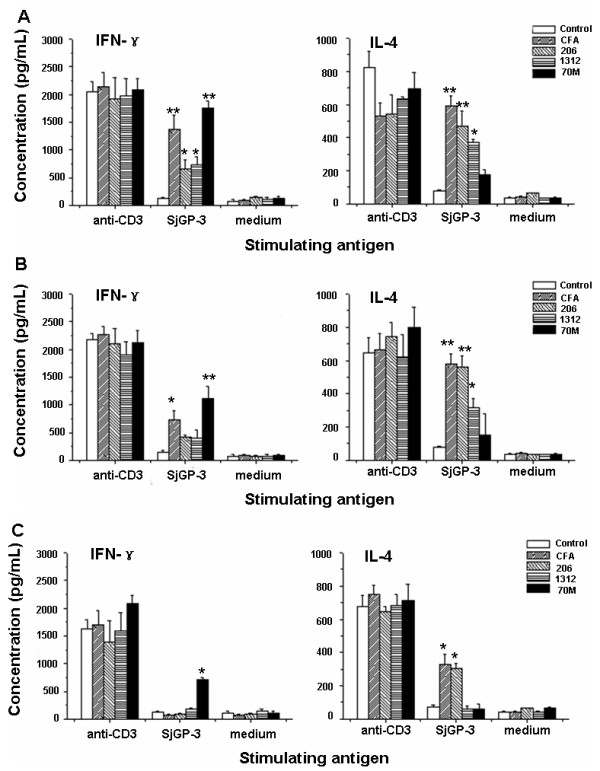
**Analysis of cytokine production**. Spleen cells were harvest from mice immunized with the SjGP-3 formulated by various adjuvants (three mice each group) on week 9 (before challenge), 12 (three weeks after cercarial challenge) and 15 (six weeks after cercarial challenge), and stimulated *in vitro *with anti-CD3, SjGP-3 protein or medium in duplicate wells respectively. The IL-4 and IFN-γ production in the culture supernatants of spleen cells was analyzed by ELISA in triplicate wells. Data correspond to the mean values ± standard deviation. **(A) **Week 9. **(B) **Week 12. **(C) **Week 15. * *p *< 0.05, ** *p *< 0.01 when compared with PBS control group.

To determine the levels of Th1/Th2 cytokines in mice immunized with SjGP-3 formulated with various adjuvants, we isolated spleen cells of the immunized mice on week 9, 12 and 15. The levels of IL-4 and IFN-γ were measured by ELISA in the supernatants of cultured splenocytes stimulated by SjGP-3 recombinant protein (30 μg/ml), anti-CD3 (1 μg/ml) as positive control or medium as the negative control. As shown in Figure 3, on week 9, significantly enhanced levels of both IL-4 and IFN-γ were observed in all adjuvant groups, except in the ISA70M group, where the IL-4 level declined significantly compared with the other adjuvant groups (Figure 3A). IL-4 levels were not significantly different between the SjGP-3/70M and the control group, indicating that immune response induced by SjGP-3/70M was towards a Th1-dominant cytokine pattern. Interestingly, this cytokine pattern generated by the SjGP-3 stimulation of the splenocytes *in vitro *could be maintained throughout parasite maturation (Figure 3B, week 12) and after onset of female worm egg production (Figure 3C, week 15). In contrast to results observed in ISA 70M group, production of IFN-γ decreased in the SjGP-3/CFA, SjGP-3/206 and SjGP-3/1312 groups on week 12 and 15 after infection, with no significant difference in cytokine levels between immunization groups and control group, with the single exception of the CFA adjuvant group on week 12. However, SjGP-3/CFA and SjGP-3/206 groups with minimal IFN-γ production presented lasting high levels of IL-4, indicating a shift in dominant cytokine profile from the mixed Th1/Th2 cytokine pattern to a Th2-dominant cytokine pattern after parasite maturation and onset of egg production.

### Effect of adjuvant on intracellular cytokine production by flowcytometry

**Figure 4:**
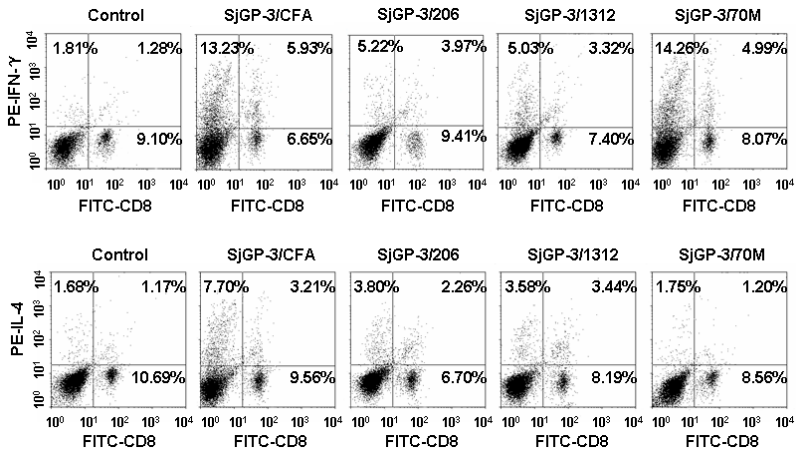
**Detection of intracellular cytokine production of splenocyte in mice by flowcytometry after immunization**. Spleen cells of mice (three mice each group) immunized with the SjGP-3 formulated by various adjuvants were harvested after three times immunization (on week 9). The isolated splenocytes from individual mice were first stimulated with PMA, ionomycin plus BFA. Splenocytes were stained extracellularly by PE-Cy5 labeled anti-CD3 and FITC labeled anti-CD8. After fixation and permeabilization, the spleen cells were divided into three tubes and stained intracellularly with PE conjugated Rat IgG1, anti-mouse IFN-γ antibody or anti-mouse IL-4 antibody, respectively. A FACScan flow cytometer with Cell Quest software was used for data acquisition and analysis. The percentage of PE positive cells in Rat IgG1 isotype control was less than 0.5% (data not shown).

To detect the percentage of CD_3_^+^CD8^- ^cells and CD_3_^+^CD8^+ ^cells in mice immunized with SjGP-3 formulations, we isolated spleen cells on week 9. As shown in Figure 4, compared with control group, the percentages of both CD_3_^+^CD8^-^IFN-γ^+ ^cells, CD_3_^+^CD8^+^IFN-γ^+ ^cells and CD_3_^+^CD8^-^IL-4^+ ^cells, CD_3_^+^CD8^+^IL-4^+ ^cells were significantly increased in mice immunized three times with SjGP-3/CFA, SjGP-3/206 or SjGP-3/1312, indicating mixed Th1/Th2 immune responses were induced. Interestingly, high percentages of CD_3_^+^CD8^-^IFN-γ^+ ^(14.26%) and CD_3_^+^CD8^+^IFN-γ^+ ^(4.99%) cells were observed in the SjGP-3/70M group, whereas there was no increase in CD_3_^+^CD8^-^IL-4^+ ^or CD_3_^+^CD8^+^IL-4^+ ^cells compared with that in control group, suggesting the immune response was polarized to Th1 profile when mice immunized with SjGP-3/70M. This was consistent with results for cytokines in the supernatant of cultured splenocytes stimulated by SjGP-3.

**Figure 5:**
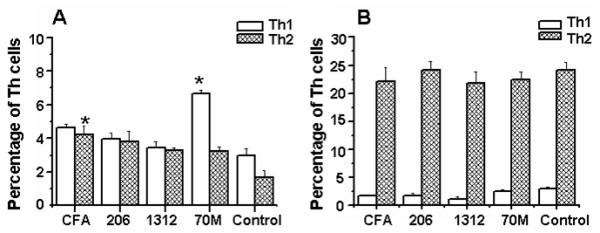
**Detection of intracellular cytokine production of splenocytes in mice by flowcytometry after cercarial challenge**. Spleen cells of mice (three mice each group) immunized with the SjGP-3 formulated by various adjuvants were harvested on week 12 (three weeks after cercarial challenge) and 15 (six weeks after cercarial challenge). The isolated splenocytes from individual mice were first stimulated with PMA, ionomycin plus BFA. Splenocytes were stained extracellularly by PE-Cy5 labeled anti-CD3 and FITC labeled anti-CD8. After fixation and permeabilization, the spleen cells were divided into three tubes and stained intracellularly with PE conjugated Rat IgG1, anti-mouse IFN-γ antibody or anti-mouse IL-4 antibody, respectively. A FACScan flow cytometer with Cell Quest software was used for data acquisition and analysis. **(A) **Week 12. **(B) **Week 15. * *p *< 0.05 when compared with PBS control group.

We also investigated percentages of CD_3_^+^CD8^- ^cells at various time points of post immunization in mice. On week 12, when the parasite was an adult but still not able to produce eggs, the Th1/Th2 cytokine profiles changed (Figure 5A). The percentage of CD_3_^+^CD8^-^IFN-γ^+ ^cells decreased to a level similar to CD_3_^+^CD8^-^IL-4^+ ^cells, with the exception of the SjGP-3/70M group, where CD_3_^+^CD8^-^IFN-γ^+ ^cells remained predominant. On week 15, after onset of egg production and maturation, the Th1/Th2 cytokine profiles were further changed (Figure 5B). The percentage of CD_3_^+^CD8^-^IL-4^+ ^cells significantly increased by > 20% while the CD_3_^+^CD8^-^IFN-γ^+ ^cells decreased in all groups, indicating development of an egg-specific polarized Th2-type immune response. The FACS dot plots of intracellular cytokine production of splenocytes after cercarial challenge were shown as an on-line supplementary figure (Additional file 1).

### Correlation of Th1-dominant response with reduction of liver eggs

**Figure 6:**
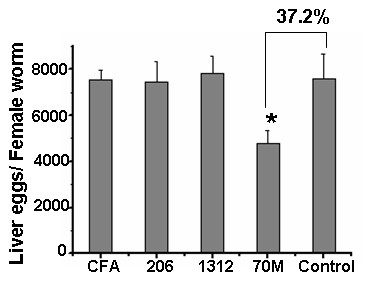
**Effect of different adjuvant on liver egg numbers per female worm**. Liver egg numbers per female worm was measured by total number of liver eggs/total number of paired female worm of individual mouse. The reduction rate of liver eggs per female worm was calculated according to the following formula: (1- mean number of liver eggs per female worm in immunized mice/mean number of liver eggs per female worm in control mice) × 100. There was a significant reduction of the number of liver eggs per female in ISA 70M groups at 34.5–37.2%, while the number of liver eggs per female was not significantly reduced in CFA, ISM 1312 and ISA 206 adjuvant groups (Exp 1: 0.6%, 1.9% and -3.2%; Exp 2: -4.1%, -3.4% and -12.5%, respectively). The number of mice per group is from 8 to 10. * *p *< 0.05 when compared with PBS control group. This picture shows one experiment representative of two independent trials with similar results.

To investigate the effect of Th1 or Th2-dominant response on protection against cercarial challenge, mice were immunized with SjGP-3 formulated in various adjuvants. As described above, the formulations with CFA, ISA 206 and ISM 1312 adjuvants induced a moderate mixed Th1/Th2 response after three vaccinations. Subsequent challenge with schistosome cercariae produced higher percentages of worm reduction (41.1%, 30.4% and 24.7%, respectively) but showed less efficacy of liver egg reduction (26.6%, 25.6% and 14.4%, respectively) in the first experiment (Table 2, Exp 1). In contrast, the ISA70M adjuvant formulation elicited a polarized Th1 response as described above but generated little effect on worm burdens (17.3% reduction) with a greater effect on egg deposition in liver (47.0% egg reduction). All results were repeatable in the second experiment (Table 2, Exp 2). Additionally, we compared the number of liver eggs per female produced in immunized mice with control group. As shown in Figure 6, the number of liver eggs per female was not significantly reduced in CFA, ISM 1312 and ISA 206 adjuvant groups. In contrast to these adjuvants, SjGP-3 formulated with ISA 70M adjuvant induced a significant reduction in the number of liver eggs per female at 37.2% and 34.5% in the first and second experiments, respectively.

## Discussion

Poor protective efficacy is one of the main obstacles to development of vaccines against schistosomes [24,25]. Although vaccination with UV-attenuated cercariae can induce potent immunity against challenge-infection in mice [26], the use of this approach is still controversial [27]. Meanwhile, all the vaccine candidates used in large animal experiments have been built on single protein antigens, which do not provide consistently sufficient protection against *S. japonicum *[24]. Polyvalent subunit vaccines containing multiple protective domains or epitopes from different antigens may be necessary for enhanced immunogenicity and protective efficacy. Chimeric protein vaccine constructs may be an approach towards this and therefore, we have constructed four fusion proteins by combining Sj26GST and paramyosin fragments. Immunization of mice with the chimeric proteins provided various levels of protection against cercarial challenge. Of the four chimeric proteins, SjGP-3 was shown to induce a high level of protection in terms of reduced worm burdens and lower liver egg counts. Moreover, immune sera to this protein interacted with both native and recombinant Sj26GST and paramyosin. Importantly, this chimeric protein provided greater protection in reduction of worm burdens and egg counts than did its individual component, Sj26GST. Therefore, SjGP-3 may be a suitable candidate for further development as a vaccine against schistosomiasis japonica.

Although it is generally agreed that a successful schistosomiasis vaccine will have the generation of an antigen-specific CD4^+ ^T cell response, it is still unclear which subset of CD4^+ ^T cells, Th1 or Th2, should be induced. Previous studies highlighted the important role of Th2-type immune response in naturally acquired resistance to infection [28-31]. However, this type of immune response also plays a key role in granuloma formation and pathogenesis of hepatic fibrosis in schistosomiasis [32]. Therefore, the possibility that vaccination may induce a Th2 response and lead to more severe disease is of concern. Other studies observed that high levels of protective immunity were related with Th1-dependent mechanisms in animals [33-35]. Recent publications showed that manipulation of the immune response for sustained Th1 response or cercarial infection of IL-4 receptor deficient mice resulted in reduced worm burdens or diminished granuloma formation [36-38]. These results imply the importance of the sustained Th1 response for anti-pathology vaccine development. In addition, radiation attenuated cercariae vaccine induced consistently high-level protection against challenge infection. Single vaccination of the irradiated cercariae elicited a Th1-dominant response and the protective mechanism likely involved cell-mediated immunity, with IFN-γ-activated macrophages and NO implicated [26,39,40].

Consistent with other findings [41,42], our results showed the correlation of Th1-type polarization with reduction of liver eggs and liver eggs per female. Several parameters indicated that the immune response was polarized to the Th1-type profile, and cellular and humeral responses were enhanced in SjGP-3/70M group: (1) IgG2a was elicited at 5- to 43-fold higher levels than in the other adjuvant groups whereas IgE titers were lower than that in all groups; (2) a Th1-dominant cytokine pattern was detected in the supernatant of splenocyte stimulated by the antigen; (3) the enhanced levels of IFN-γ were lasting, whereas the IL-4 levels were similar to the control group; and (4) consistent with the cytokine pattern, staining of splenocytes and flow cytometric analysis showed that CD3^+^CD8^-^IFN-γ^+ ^and CD3^+^CD8^+^IFN-γ^+ ^cells in this group were significantly increased, whereas CD3^+^CD8^-^IL-4^+ ^and CD3^+^CD8^+^IL-4^+ ^cells were not increased compared with control group. Since schistosome eggs are the principal cause of pathology in schistosomiasis, reduction in liver egg may be a more meaningful endpoint than adult worm burdens. Most researches on schistosomiasis vaccine focused on development of anti-worm vaccines. However the reduction of worm burden induced by such vaccines can't consistently supply 40% protection in challenge experiments, a level that the WHO believes necessary for protection against schistosomiasis [5,25]. Thus, development of an effective vaccine that could significantly reduce female egg production so that tissue egg burdens are lowered to a level where little morbidity and mortality occurs and parasite transmission is limited. The immune mechanism underlying this protection is unclear. In this study, we observed cercarial challenge of mice with the polarized Th1-type response resulted in significantly reduced the number of liver eggs per female (34.5–37.2%), whereas the number of liver eggs per female was not reduced in the other groups. This might suggest that anti-fecundity protective immunity was induced by deviating immune response towards Th1 polarization. However, attenuated cercariae did not induce such anti-fecundity effect in immunized mice even though this immunization stimulated strong Th1 response [42]. Thus, it is necessary to analyze whether SjGP-3/70M induced anti-fecundity immunity is undermined in IFN-γ-/- mice.

SjGP-3 formulated in various adjuvants induced different types of immune responses. However, the SjGP-3-induced immune response patterns were significantly modulated by parasite antigens. The Th1-dominant pattern in the SjGP-3/70M group was modulated after cercarial infection, revealing the CD3^+^CD8^-^IL-4^+ ^cells expand dramatically after parasite maturation. Moreover, the immune response was further polarized to a Th2-type profile after the onset of egg production by the parasites and their maturation. This data suggested that the SjGP-3-induced polarized Th1 response failed to prevent the shift from Th1 to Th2 transition normally occurring in infected mice after the onset of female worm egg production. Cytokine production in culture supernatants of spleen cells stimulated by SjGP-3 did not appear to change to the intracellular cytokine patterns after cercarial challenge, but this may be due to the inability of the detection methodology to detect cytokines secreted by anything other than SjGP-3-specific lymphocytes. We noticed the discrepancy of cytokine pattern changes between SjGP-3 stimulation and anti-CD3 stimulation. When the cells were stimulated by anti-CD3 at an antigen non-specific fashion for 72 hrs, all subsets of Th cells including Th0, Th1 and Th2 would be activated and corresponding cytokines would be produced. Therefore, when the polarized Th1 cells in the SjGP-3/70M group were activated with anti-CD3, the Th0 cells mixed in them will also be activated by anti-CD3 to generate IL-4. This IL-4 will create difficulties in the determination of Th1 phenotype. On the other hand, the large amount of IFN-γ generated by Th0 cells from control group made us hard to see an increased IFN-γ production in SjGP-3/70M group as that found in antigen specific stimulation fashion. These two factors collectively made us fail to demonstrate Th1 development in anti-CD3 stimulation. Concerning the intracellular cytokine staining results of SjGP/70M group shown in Figure 4 and Figure 5A which actually showed Th1 phenotype even with antigen non-specific stimuli PMA and ionomycin, our explanation is that the activation time only lasted for 6 hrs in vitro. This short time non-specific stimulation will only reactivate those Th cells which were activated and polarized in vivo by SjGP-3/70M. For this reason, Th1 phenotype demonstrated by FACS data was in consistent with the Th1 phenotype demonstrated by measurement of cytokine production stimulated by antigen in vitro.

*S. japonicum *is a zoonotic parasite. Domestic animals, especially water buffalo, are the major reservoirs for *S. japonicum *infection in China and responsible for 75% of human transmission [43]. Mathematical modeling of *S. japonicum *transmission dynamics in China revealed that an anti-fecundity bovine vaccine to a level at 75% efficacy ensured the reduction of parasite in human population [44]. Although a vaccine specific for use in humans may be developed in the future, development of a veterinary vaccine may be a practical interim strategy. Few veterinary adjuvants have been identified for schistosomiasis vaccine development; therefore, selection of appropriate adjuvants is an important first step in this process. Here, we compared the effect of four adjuvants (CFA, ISA 206, IMS 1312 and ISA 70M) combined with SjGP-3 in mice challenge experiments. ISA 206, IMS 1312 and ISA 70M are known as veterinary adjuvants and represent three different kinds of emulsions, the water-in-oil (ISA70M), the water-in-oil-in-water (ISA 206) and the nanoparticles of IMS 1312 [45,46]. We found the use of adjuvant exhibited a profound improvement on the protection efficacy of SjGP-3 in *S. japonicum *challenge in mice. Of the novel adjuvants used in this study, ISA70M comprises a high grade injectable mineral oil and an extremely refined emulsifier obtained from mannitol and purified oleic acid of vegetable origin. This adjuvant is a modified version of a successfully used animal adjuvant ISA 70 [47]. Interestingly, the SjGP-3 antigen when formulated with this adjuvant induced enhancement in both humoral and cell-mediated immune response, by developing a polarized Th1 response resulting in significant reduction of liver eggs.

## Conclusion

In conclusion, construction of polyvalent subunit vaccine was capable to enhance immunogenicity and protection efficacy against schistosomiasis japonica. There was correlation of the polarized Th1 response with reduction of liver egg burdens, indicating the immune deviation strategy for schistosomiasis japonica vaccine development. These data support further investigation of the SjGP-3 formulation with ISA 70M as a potential vaccine for animal use.

## Competing interests

The authors declare that they have no competing interests.

## Authors' contributions

XDX, DMZ and WQP participated in the design of the study. XDX, WS, JJZ and LHS performed the experiments. XDX, QFZ, XYX and WQP participated in the statistical analysis. XDX and WQP drafted the manuscript. All authors read and approved the final manuscript.

## Pre-publication history

The pre-publication history for this paper can be accessed here:

http://www.biomedcentral.com/1471-2334/9/54/prepub

## Supplementary Material

Additional file 1 **Figure S1**. The FACS dot plots of intracellular cytokine production of splenocytes after cercarial challenge. **(A)**: Week 12; **(B)**: Week 15. Click here for file
