# Supplementary figures and images for: A Schistosoma japonicum chimeric protein with a novel adjuvant induced a polarized Th1 immune response and protection against liver egg burdens
Source: BMC Infect Dis. 2009 May 6;9:54. doi: 10.1186/1471-2334-9-54 (PMC2685138; doi:10.1186/1471-2334-9-54)

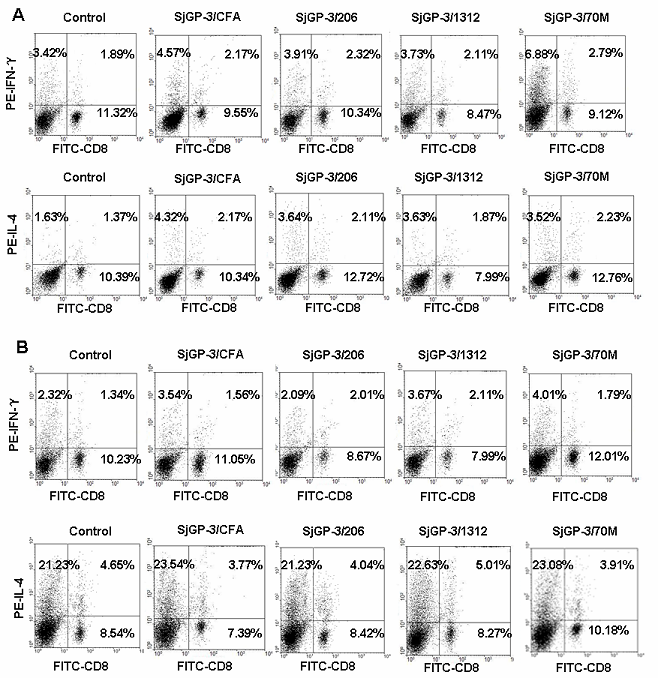

Supplement: Additional file 1 — Figure S1. The FACS dot plots of intracellular cytokine production of splenocytes after cercarial challenge. (A): Week 12; (B): Week 15. [file 1471-2334-9-54-S1.tiff]
